# Enhanced sampling without borders: on global biasing functions and how to reweight them

**DOI:** 10.1039/d1cp04809k

**Published:** 2021-12-14

**Authors:** Anna S. Kamenik, Stephanie M. Linker, Sereina Riniker

**Affiliations:** Laboratory of Physical Chemistry, ETH Zurich Vladimir-Prelog-Weg 2 8093 Zurich Switzerland sriniker@ethz.ch

## Abstract

Molecular dynamics (MD) simulations are a powerful tool to follow the time evolution of biomolecular motions in atomistic resolution. However, the high computational demand of these simulations limits the timescales of motions that can be observed. To resolve this issue, so called enhanced sampling techniques are developed, which extend conventional MD algorithms to speed up the simulation process. Here, we focus on techniques that apply global biasing functions. We provide a broad overview of established enhanced sampling methods and promising new advances. As the ultimate goal is to retrieve unbiased information from biased ensembles, we also discuss benefits and limitations of common reweighting schemes. In addition to concisely summarizing critical assumptions and implications, we highlight the general application opportunities as well as uncertainties of global enhanced sampling.

## Abstract 2

Global enhanced sampling techniques bias the potential energy surface of biomolecules to overcome high energy barriers. Thereby, they aim to capture extensive conformational ensembles at comparably low computational cost.

## Graphical abstract

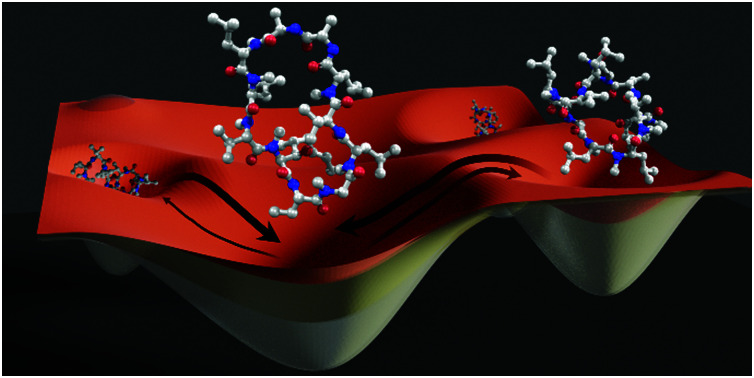

## 1 General introduction

Biomolecules in solution constantly fluctuate within an ensemble of conformational states with varying probability.^1^ Each of these conformational states exhibits (slightly) altered biophysical properties and provides different opportunities for interactions with surrounding molecules.^2–5^ The dynamic nature of biomolecules is thus essential to both fundamental and applied research, *e.g.* for drug discovery and lead optimization.^1,3,6^ However, even after several decades of research in this area, the complexity and longevity of the conformational rearrangements of biomolecules still poses substantial challenges for computational and experimental methods.^4,7^ The ideal technique would be a “molecular camera”, which records the time evolution of the motion of a single molecule in atomic resolution. Despite massive progress in the field of experimental integrative modeling, the tool that currently comes closest to this ideal of a molecular camera is molecular dynamics (MD) simulations.^8,9^ The theoretical framework as well as the implementation of MD simulations is built on numerous approximations to reduce the computational costs to a tractable level, which limits the accuracy of the resulting dynamic models.^10–12^ These approximations can be broadly divided into two categories: (i) inaccuracies in the underlying force field, and (ii) uncertainties due to limited phase-space exploration. Within this review, we will focus on the latter, which is traditionally also referred to as the “sampling problem”.

A typical biomolecular system is characterized by a myriad of degrees of freedom, resulting in practically innumerable conformational and configurational states. This complex phase space translates into a free-energy surface that is vast and rugged. MD simulations offer the possibility to explore such free-energy landscapes with a resolution of nanometers and femtoseconds. However, in practice the system often gets trapped in a (local) minimum as high barriers to neighboring configurational states impose slow transition rates. With dedicated state-of-the-art hardware or exa-scale cloud-computing infrastructure, motions on the millisecond timescale can be observed for biomolecular systems of considerable size.^13–15^ Unfortunately, the general access to such supercomputing systems is limited. An alternative to brute force high-performance computing is to speed up the sampling process using enhanced sampling strategies.

Many different methodologies that fall into the category of enhanced sampling have been developed over the past decades (for previous reviews we refer the reader to ref. 16 and 17). Some of the most popular enhanced sampling strategies are pathway-dependent, meaning they rely on the definition of low-dimensional order parameters, also called reaction coordinates or collective variables (CVs). Methods such as local elevation^18^ or metadynamics,^19^ umbrella sampling^20,21^ or targeted MD^22^ increase sampling efficiency in a simulation by applying a bias along the selected CV. Consequently, identifying representative CVs is critical to the success of pathway-dependent enhanced sampling techniques, but reducing the complex dynamics of biomolecules to a few interpretable dimensions is far from trivial.^23^ Substantial research efforts are currently invested in the optimization and automatization of selecting appropriate CVs, *e.g.* with the aid of machine learning.^24–26^ Given relevant CVs, pathway-dependent methodologies can perform strikingly well, for example in modelling the activation of voltage-sensing domains of ion channels,^27,28^ the estimation of ligand *k*_off_ rates,^29^ or membrane permeation probability calculations.^30,31^

Despite these successes, for many interesting biomolecular systems it is not straight-forward to derive a small number of representative observables as CVs. For example, when we simulate cyclic peptides in apolar environments, we usually observe one (or a few) well-defined “closed” structures. These closed conformational states can often be easily represented, *e.g.*, *via* intramolecular hydrogen bond formation.^32,33^ However, when we study the same system in a polar environment, defining a unique representation immediately becomes more difficult. The ensembles of cyclic peptides in polar environments are generally much more diverse, and observables such as intramolecular hydrogen bonds or the radius of gyration fail to distinguish the conformational states. Other scenarios, which are challenging for CV-based pathway-dependent methods, include studies with the specific aim of identifying the most flexible domains of a biomolecule,^34–36^ or of discovering novel cryptic or allosteric binding sites.^37–39^ Whenever the goal is to explore and compare local flexibility patterns within one biomolecular system, a pathway-dependent bias should be avoided as it inherently steers the results towards the user-defined reaction coordinate.

Fortunately, also pathway-independent enhanced sampling techniques have been developed, which do not require the definition of CVs. Methods following the principles of hyperdynamics^40^ or parallel tempering^41,42^ add global biasing energies that act on the entire system simultaneously. Here, we provide an overview of currently available pathway-independent enhanced sampling methods, which we broadly categorize by whether the bias is defined *via* the potential or kinetic energy function. For each of the methodologies we describe in the following sections promising results that have been reported for various scientific problems. However, each approach also has its limitations. As we summarize benefits and pitfalls, we explain what can and cannot be expected from the different methods. Furthermore, we discuss the general and central challenge of extracting unbiased thermodynamic and kinetic information from biased ensembles. This process, typically referred to as reweighting, is in practice often decisive for the applicability of biasing methods.

## 2 Turning up the heat: biasing the kinetic energy

**Fig. 1:**
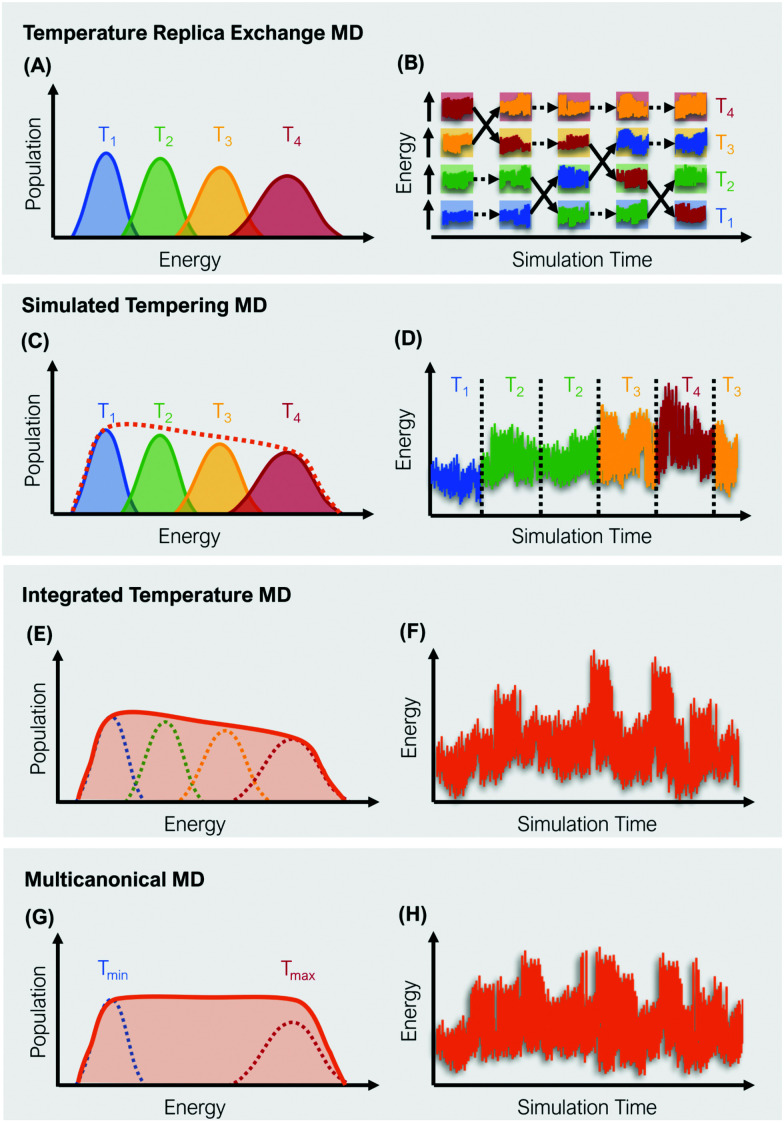
Schematic representation of four global enhanced sampling techniques, where the bias is defined *via* the kinetic energy of the system: Temperature replica exchange MD (A and B), simulated tempering MD (C and D), integrated temperature MD (E and F) and multicanonical MD (G and H). The left column (A, C, E and G) illustrates the sampled energy distributions, while the right column (B, D, F and H) displays the energy as a function of the simulation time.

A straight-forward way to increase the velocity of motions in a simulated system is to elevate its temperature, *i.e.*, to introduce a kinetic bias. At higher temperatures, the transition rates between local minima increase, thus a larger phase space can be explored in less computational time. Simulations at room temperature on the other hand ensure thorough sampling within the minima.^43,44^ The tempering approaches described below follow the same fundamental idea: Simulations at high and low temperatures are combined based on an energetic criterion, which retains the canonical ensemble (Fig. 1). Through this, conformational sampling is achieved more efficiently and more reliably than with conventional – single temperature – MD simulations. The practical implementation of this idea differs, however, greatly between individual enhanced sampling strategies, which we will outline in detail in the following paragraphs.

### 2.1 Temperature replica exchange MD

In temperature replica exchange MD (T-REMD), also referred to as parallel tempering, multiple simultaneous simulations of identical systems are performed at varying temperatures.^41,42^ Exchanges between neighboring replicas are attempted at defined time intervals and accepted or rejected based on an energetic criterion, which retains the canonical distribution (Fig. 1A and B).^45^ Typically, this routine is based on a Metropolis criterion, where the exchange probability *P*(*T*_*k*_ → *T*_*l*_) depends on the reference temperature of two replicas (*T*_*k*_, *T*_*l*_) and their potential energies (*V*_*k*_, *V*_*l*_):^46, 47^1
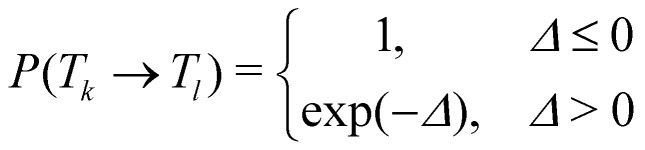

2 *Δ* = (*β*_*k*_ − *β*_*l*_)(*V*_*k*_ − *V*_*l*_),

with *β*_*k*_ = 1/*k*_*B*_*T*_*k*_, *β*_*l*_ = 1/*k*_B_*T*_*l*_ being the inverse of the temperature *T*_*k*_ and *T*_*l*_ multiplied by the Boltzmann constant *k*_B_. The nature of this approach thus requires a computational setup, where multiple simulations can be performed in parallel with sufficiently fast and frequent communication between the computing nodes.^43^ While the parallelization posed a limitation for the applicability of the approach in the past, nowadays parallel computing is widely available with modern computing environments. However, the challenge increases for large biomolecular systems as the number of required replicas is estimated to increase with *N*^1/2^ for a system with *N* degrees of freedom.^48^ Nevertheless, assuming that the required high-performance computing environment is available, the main question remaining is how to choose the number and spacing of replicas and the overall temperature range. Various, mostly iterative schemes have been proposed to optimize the choice of these parameters.^49,50^ A quite popular tool to estimate the replica distribution is the temperature generator for REMD-simulations, introduced and hosted by David van der Spoel and co-workers.^47,51^ Their algorithm estimates the number and spacing between T-REMD replicas based on system size, temperature range, and a user-defined exchange probability (a practical rule of thumb is to choose an acceptance rate above 0.2 to 0.3.^52^) These predictions are, however, based on a certain test setup and should be re-evaluated for the user-specific combinations of MD engine and force field. For a detailed practical guide on how to run T-REMD simulations, we refer the interested reader to ref. 52.

Compared to serial simulated tempering (ST) simulations, T-REMD simulations have been found to converge slower at a higher computational cost,^53^ although depending on the studied system and simulation setup, T-REMD simulations could theoretically outperform ST simulations in terms of wall time. In practice, the use of T-REMD is notably more popular. This can most likely be attributed to its straight-forward implementation and the fact that no weighting factors need to be optimized.^53^ Furthermore, T-REMD simulations result in extensive sampling at different temperatures, which can provide valuable information beyond enhanced conformational sampling. Some of the most remarkable studies working with T-REMD include the first study of Sugita and Okomoto on the folding of Met-enkephalin,^42^ which was followed by numerous works using T-REMD to further elucidate the protein folding problem.^54–58^ Moreover, T-REMD has greatly aided the interpretation of experimental data from various sources.^59–61^ T-REMD simulations have also shown promising results in the area of cyclic peptides, generating ensembles that agree well with NMR interproton distances.^62^ In the study by Wakefield *et al.*,^62^ the authors were furthermore able to rationalize the varying binding affinities of the cyclic peptides through conformational pre-organization captured in T-REMD. In a different study, solvent induced conformational changes could be observed for cyclic tetrapeptides using T-REMD.^63^ Additionally, T-REMD simulations have shown valuable benefits in the parametrization of residue-specific force fields.^64^

### 2.2 Simulated tempering

ST simulations, also called serial tempering,^44^ follow a similar sampling strategy as T-REMD. In ST simulations, temperature switches are also accepted or rejected based on an energetic criterion. The major difference in the ST setup is, however, that only one continuous simulation is performed and the Hamiltonian becomes dependent on the system's reference temperature *T*_*i*_ in this single simulation (Fig. 1C and D).^65,66^ Let's consider a system where *H*(*r*) is the Hamiltonian of configuration *r*. As we choose a discrete set of temperatures *T*_1_ < … < *T*_*K*_, we define a generalized Hamiltonian^44^ to run the ST simulation:

3 ℋ(*r*,*k*) = *β*_*k*_*H*(*r*) − *g*_*k*_,

here, the index *k* is referring to the temperature range and thus can take values from 1 to *K*. The logarithmic weight corresponding to the temperature *T*_*k*_ is denoted as *g*_*k*_. Consequently, the generalized partition function *Z* can be written as,4
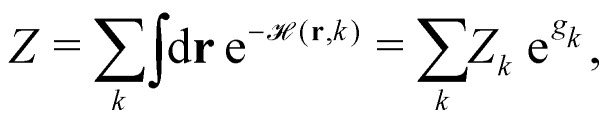
with *Z*_*k*_ being the partition function at *β*_*k*_. This notation highlights that the generalized ensemble combines the canonical ensembles at each temperature using the weighting coefficients {*g*_*k*_}. With a user-defined frequency, attempts are made during the simulation to alter the system temperature *T*_*k*_ to a new trial temperature *T*_*l*_, which is taken from the discrete set of selected temperatures. Whether or not the change is accepted is evaluated based on an energetic criterion, which is specifically designed to maintain the canonical distribution. Similar to T-REMD simulations, the acceptance probability for *k* → *l* is defined by *min*(1,e^−Δℋ_*k*→*l*_(*r*)^),^66^ where

5 Δℋ_*k*→*l*_(**r**) := ℋ(**r**, *l*) − ℋ(**r**, *k*) = (*β*_*l*_ − *β*_*k*_) *H*(**r**) − (*g*_*l*_ − *g*_*k*_).

Consequently, unbiased statistics of each temperature are collected for ST (as well as for T-REMD simulations), which do not need additional reweighting if analyzed individually. Achieving uniform sampling across the selected set of temperatures critically depends on the choice of the weighting coefficients *g*_*k*_. Optimization of these weights (and the automatization of it) is thus the main challenge in working with ST simulations.^67^ In practice, this is often a tedious task, which requires numerous short trial simulations as the weights are not known a priori.^44,68^ Nonetheless, ST simulations have been found to be quite robust across various computing environments as they only require a single computing node. The approach has already facilitated several studies which explore the free-energy landscapes of biomolecules (*e.g.* BPTI, Villin, Trp-cage, or fast folding WW-domain peptides) at low computational cost with speedups of several orders of magnitude.^68,69^ Further prominent examples for the application of ST simulations include folding dynamics of multiple mini-proteins in explicit solvent^68,69^ and Alzheimer related peptide aggregation.^67^

### 2.3 Integrated temperature sampling

Integrated temperature sampling (ITS) introduces a sum-over-temperature non-Boltzmann factor, which is essentially a linear combination of Boltzmann distributions at different temperatures.^70,71^ This means that the simulation is performed on a temperature-biased effective potential *Ṽ*(*r*), which is defined in the following manner for each configuration *r*:6
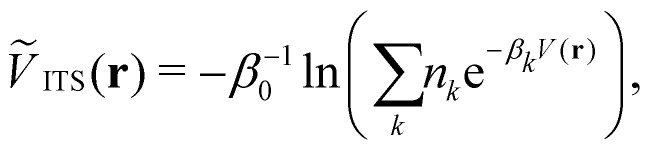
where *β*_0_ corresponds to the the desired inverse reference temperature and {*β*_*k*_} denotes the the selected inverse temperature range. {*n*_*k*_} are the weighting coefficients, which determine the contribution of the potential energy *V*(*r*) at each inverse temperature *β*_*k*_. Typically, it is best to sample the full temperature range uniformly. However, in theory it is straight-forward to focus the sampling to selected temperature regions using {*n*_*k*_}.^72^ Hence, ITS bears similarities to enveloping distribution sampling,^73^ as it combines multiple potentials (at different temperatures) to sample one generalized (non-Boltzmann) distribution. The resulting effective potential thus facilitates efficient phase-space exploration across a chosen temperature range within a single simulation (Fig. 1E and F).^45,72^ The main challenge in ITS is to identify suitable coefficients {*n*_*k*_}, which can be estimated in an iterative procedure during the simulation.^72,74^ This central process is fast for comparably simple systems, but can become much more difficult for large-scale biomolecular systems.^70^ This potential limitation is balanced against the advantageous features of the method, *i.e.* that the convergence behavior of ITS has been observed to be superior to other global enhanced sampling techniques and that it is computationally substantially more efficient than the related T-REMD in terms of CPU time.^45^

### 2.4 Multicanonical molecular dynamics

Multicanonical MD (McMD) simulations aim to uniformly sample the potential-energy surface (PES) between high temperature and low temperature regions (Fig. 1G and H).^75,76^ In other words, the aim is to derive a flat probability distribution function *P̃*_McMD_ of the potential energy *V*:^75^7
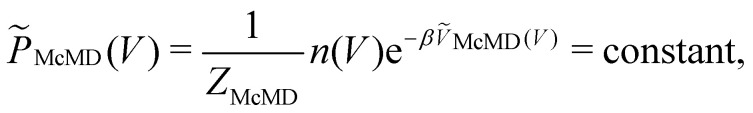
where the partition function in the multicanonical ensemble is defined as 
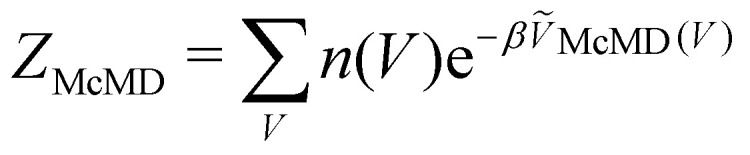
, *n*(*V*) is the density of states, and *Ṽ*_McMD_(*V*) is the effective potential of the McMD simulation. *Ṽ*_McMD_(*V*) is not known *a priori* and needs to be determined from a preceding conventional MD simulation as a function of its unbiased potential energy, *V i.e.*,^77^

8 *Ṽ*_McMD_(*V*) = *V* + *k*_B_*T*_0_ ln(*P*(*V*, *T*_0_)),

with *P*(*V*, *T*_0_) being the probability distribution of the unbiased potential energy *V* and the selected temperature *T*_0_. This initial temperature *T*_0_ is typically set to comparably high values to cover a sufficiently broad energetic space. However, the resulting *Ṽ*_McMD_(*V*) usually cannot be used as the final effective potential for productive McMD runs. It rather serves as the first input for an iterative refinement scheme that optimizes *Ṽ*_McMD_(*V*) for a particular system under study.^77^ The aim of this refinement is to converge to that flat probability distribution function between a chosen temperature range [*T*_min_, *T*_max_], where *T*_min_ is generally chosen to be slightly below room temperature and *T*_max_ is in the range of 700 K to 1000 K.^78^ Since its first introduction by Berg and Neuhaus in 1992, who applied the McMD algorithm to study the two-dimensional Potts system,^79^ McMD has found broad usage in the biomolecular simulations community.^77,80^ In particular for investigations on the dynamics of (bio-) pharmaceutical systems such as antibodies^81,82^ and cyclic peptides,^76,83^ McMD was successfully applied to bypass high energetic barriers.

## 3 Flooding valleys and shaving peaks: biasing the potential energy

More common than changing the kinetic component of the Hamiltonian is the introduction of a bias to the potential energy. Different algorithms have been developed, which decrease the barriers between conformational states either by “filling up” the minima or flattening the maxima of the PES. The varying benefits and limitations of the approaches described in the following paragraphs mostly stem from differences in the functional form of the implemented bias.

### 3.1 Hyperdynamics

The general idea of distorting the PES with a global biasing potential was first introduced by Arthur Voter in 1997.^40^ The initial implementation of the approach required the calculation of the Hessian matrix to identify transition states, which inherently limited its applicability to relatively small systems. In further development, Hamelberg *et al.*^84^ reformulated the approach for large solvated biomolecular systems using a more simplistic biasing potential. From then on the approach became known as accelerated MD (aMD) simulations.

In aMD simulations, a biasing term Δ*V*_aMD_(**r**) is added to the potential energy *V*(**r**), whenever it is below a certain threshold *E*. The effective potential *Ṽ*_aMD_(**r**) can thus be written as,9
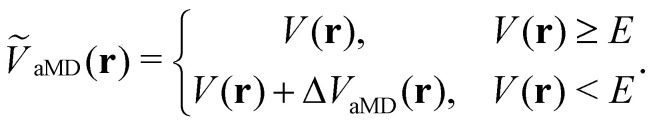


**Fig. 2:**
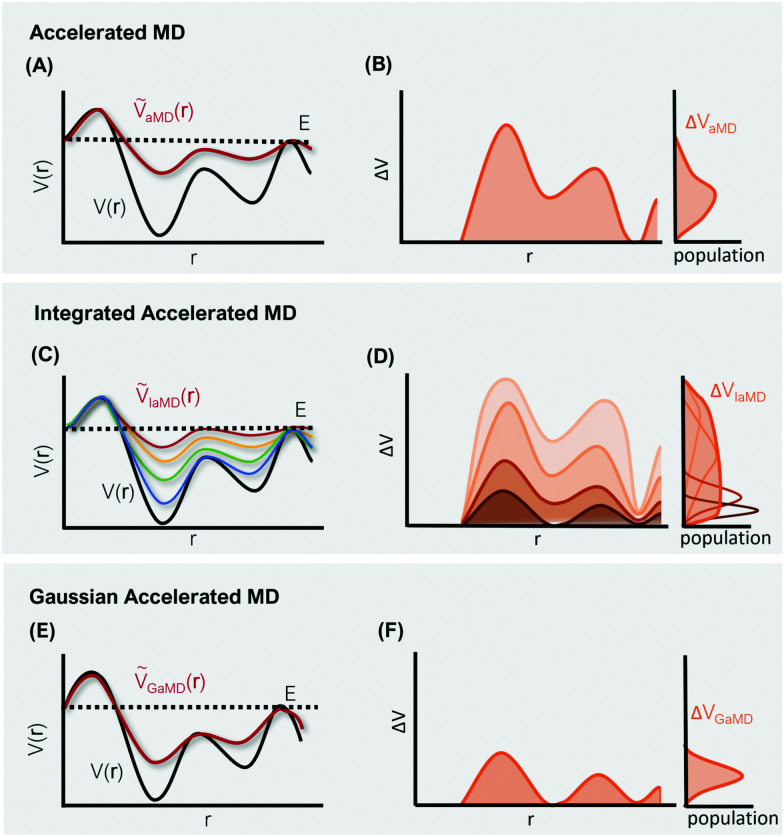
Schematic representation of three different hyperdynamics implementations: accelerated MD (A and B), integrated accelerated MD (C and D), Gaussian accelerated MD (D and E). The left column (A, C and E) illustrates the smoothing of the PES along a selected reaction coordinate *r*. The right column (B, D and F) displays the corresponding distributions of the boosting potential Δ*V* for each implementation.

In the initial formulation of aMD, the biasing term Δ*V*_aMD_(**r**) – typically referred to as boosting potential – itself is defined as,10
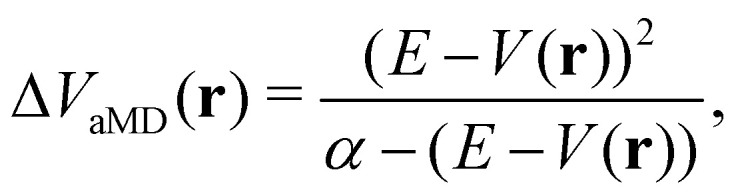
where *α* is the so-called tuning or acceleration parameter, which determines the smoothness of the effective potential *Ṽ*_aMD_(**r**) (Fig. 2). Hence, the magnitude of the added biasing potential increases when the difference between the threshold energy *E* and the unbiased potential *V*(**r**) is large. This means that the minima are elevated, which in turn decreases the height of the barriers between neighboring conformational states. From a comparison to a millisecond trajectory of the bovine pancreatic trypsin inhibitor (BPTI), it has been estimated that aMD can result in an impressive speed-up of three orders of magnitude.^85, 86^ This speed-up is, however, dependent on system size and most importantly also on the chosen acceleration parameters *E* and *α*.

Since their introduction in 2004, aMD simulations have been employed to study a broad range of biomolecules. Some of the most notable applications include the work of Miao *et al.*,^87,88^ in which the free-energy surface of G-protein-coupled receptors was elucidated, as well as the investigation from Markwick *et al.*,^89^ where aMD predicted motions in protein GB3 on the millisecond timescale in agreement with NMR measurements. Generally, aMD has shown great potential in predicting, complementing, and refining NMR data for numerous biomolecular systems.^86,90–94^ In particular, aMD simulations have also been shown to produce reliable ensembles of macrocycles and cyclic peptides,^94^ which can be further utilized for drug optimization.^95,96^

The central challenge when working with aMD is to select appropriate values for the two acceleration parameters *E* and *α*. In double boost aMD,^97^ the variant of aMD that is probably most-widely used for biomolecules, all particles in the system are biased together with an extra boost on the dihedral terms. For this setup, four parameters need to be defined, *i.e.* threshold energy and smoothing parameters for both the dihedral terms (*E*_dihed_ and *α*_dihed_) and the total potential energy (*E*_texttotal_ and *α*_texttotal_). These values are typically derived from short conventional MD simulations based on empirically derived formulae that take into account the average total energy 〈*V*_total_〉, the average dihedral energy 〈*V*_dihed_〉, and the number of atoms *n*_atom_ and number of residues *n*_res_, respectively.^85,98^ To derive the dihedral biasing potential the following equations are commonly applied:

11 *E*_dihed_ = 〈*V*_dihed_〉 + 4·*n*_res_

12
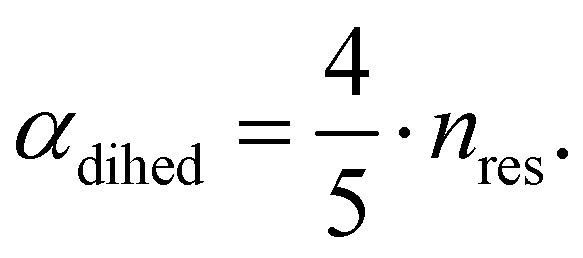


For the calculation of the total energy biasing potential, parameters can be calculated *via*13
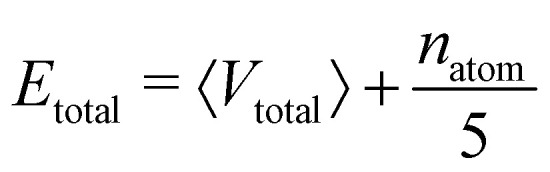
14
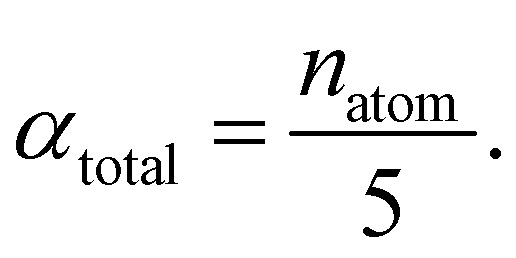


Despite these rules of thumb, choosing appropriate values for the acceleration parameters is far from trivial as the introduced bias should ideally increase sampling speed substantially without flattening the PES too severely. In our experience, it is usually worthwhile to start multiple short aMD simulation with different sets of parameters to assess the impact of the bias on the observed motions. If the PES becomes too distorted, the simulation mostly visits irrelevant high-energy states, which for proteins typically means irreversible unfolding. The latter scenario is probably one of the most dreaded risks of enhanced sampling in general, although seldom discussed in the literature. To circumvent the issue of manually selecting optimal parameters, a combination of aMD and replica exchange simulations (RE-aMD) has been proposed.^99,100^ Despite the advantages of RE-aMD, this approach has not found a great echo within the community. This might be due to the fact that RE-aMD requires replicas to be simulated in parallel, while one of the main selling points of the original aMD algorithm was that it can be carried out on a single computing node. This was a particularly intriguing feature in the early 2000s, when access to high-performance computing facilities was more scarce. Although the advances in computer power since then have worked in favor of parallel simulation techniques, the full potential of the RE-aMD approach has not yet been exploited, probably also due to several other intriguing advancements of aMD.

One of these more recent adaptations of the methodology is called integrated aMD (IaMD), which combines multiple aMD simulations with varying acceleration parameters into one trajectory by following the principle of ITS (Fig. 2).^101–103^ Here, the total boosting potential is defined as,15
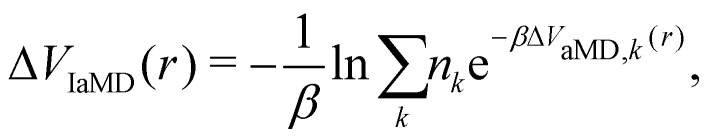
where Δ*V*_aMD,*k*_(*r*) is the standard aMD boosting potential as defined in eqn (10) for the *k*th set of acceleration parameters. The main challenge with IaMD is that – similar to ITS – the weighting coefficients {*n*_*k*_} need to be optimized in addition to the parameters {*E*} and {*α*}. A highly compelling argument for IaMD is, however, the convergence speed-up of up to three orders of magnitudes compared to aMD simulations, which has been reported for different fast folding proteins.^45,102^ This advantage arises from combining multiple biasing potentials into one, which circumvents the problem of oversampling high-energy states. Also for large biomolecular systems, such as the RNase P holoenzyme in complex with pre-tRNA, IaMD simulations have provided valuable mechanistic insights.^104^ Further variations of the aMD approach include adaptive aMD,^105,106^ where the threshold energy is reevaluated and adjusted on-the-fly during the simulation, or “lowering-barrier” aMD,^107^ where the biasing function directly acts on the maxima instead of the minima of the PES.

The probably most notable advancement of the aMD approach is termed Gaussian aMD (GaMD).^108^ The central idea in GaMD simulations is to inherently restrict the boosting potential in such a way that a Gaussian distribution is obtained (Fig. 2),16
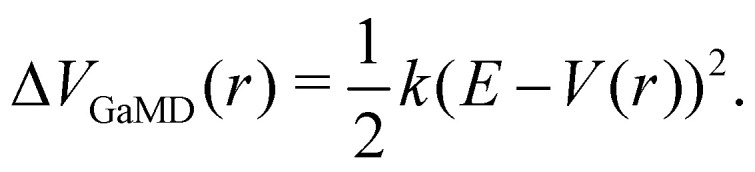


Again, this boosting potential is only applied when the system's potential energy is below a defined threshold energy *E* (see eqn (9)). In standard aMD simulations, the distribution of the boosting potential is often spread across a large energy range from tens to hundreds of kJ mol^−1^ in a non-Gaussian manner.^109^ This can lead to slow convergence as low-energy states are not sufficiently sampled, and additionally introduces severe difficulties for the subsequent reweighting procedure (see Section 4).^45^ By restricting the distribution of the boosting potential in GaMD, both of these issues can be circumvented. Nevertheless, also GaMD simulations require the definition of several parameters. These can again be optimized in an automated manner based on a short simulation preceding the production run. In this parameter optimization scheme, the threshold energy *E* is typically set to the system's maximum potential energy (while it can be freely chosen in aMD).^108^ The average boosting potential as well as its standard deviation have been shown to be lower compared to standard aMD.^108^ With the aid of GaMD simulations, large biomolecular systems have been studied, such as the CRISPR-Cas9 system,^110^ allergen-antibody complexes,^111^ or T-cell receptors binding to peptide-MHC complexes.^112^ For more details on the methodology and applications of GaMD, we recommend a recent and comprehensive review from the Palermo and Miao groups in ref. 113.

### 3.2 Hamiltonian replica exchange

Hamiltonian replica exchange MD (H-REMD) in principle also includes T-REMD, as temperature scaling inherently affects the full Hamiltonian.^48^ However, the term H-REMD is typically used to refer to methods, which alter the Hamiltonian *via* the potential-energy contribution. The replica exchange mechanism works as described for T-REMD, yet there is more freedom in the form of the implemented bias. This bias can act on selected force-field terms,^114–117^ or on the full energy function.^100,118,119^ One prominent example of a H-REMD approach is replica exchange with solute scaling (REST2).^120^ In the preceding version of this approach (*i.e.* replica exchange with solute tempering (REST1)), both the temperature and the potential energy vary between replicas.^120,121^ REST2 simulations, on the other hand, are carried out at a constant temperature *T*_0_, while the potential energy of each replica *k* is scaled *via*,17
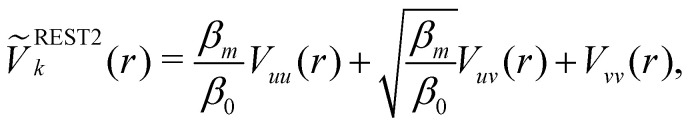
where *V*_*uu*_(*r*) is the solute–solute interaction energy, *V*_*uv*_(*r*) is the solute–solvent interaction energy, *V*_*vv*_(*r*) is the solvent–solvent interaction energy, and *β*_*m*_ = 1/*k*_b_*T*_m_. The main advantage of REST2 over REST1 and T-REMD is that it requires a smaller number of replicas and it converges faster, as shown for the test systems Trp-cage and *β*-hairpin.^120^ In our own work, we have used H-REMD to generate diverse conformational sets for cyclic peptides with scaled dihedral potentials.^32,122,123^ Other implementations that truly act on the full potential-energy function include the above mentioned RE-aMD^99,100^ as well as RE-gaMD.^119^ In both cases, the replica exchange setup bypasses the issue of choosing optimal acceleration parameters. The approaches promise enhanced sampling of all degrees of freedom while retaining sufficient sampling of low-energy states. Example applications are the folding dynamics of mini-peptides,^100^ and the dynamics of the HIV protease.^100,119^ Some practical challenges of all these H-REMD schemes are, however, the setup with the parallel replicas (*i.e.* considerable computational demand) as well as the choice of range and distribution of the replicas.

## 4 Reweighting

The purpose of MD simulations is typically to model the dynamic behavior of a system at experimental or physiological conditions. Yet, the bias introduced with enhanced sampling techniques – be it on the kinetic or potential energy - distorts the free-energy landscape and consequently does not allow direct comparison with experimental data.^124^ However, as the form and extent of the biasing potential is known at any given simulation step, the unbiased information can be retrieved using reweighting schemes.^125, 126^

Thermodynamic quantities (*e.g.* free-energy differences or stationary distributions) are usually more easily accessible than kinetic information (*e.g.* transition rates), which are particularly challenging to recover.^127,128^ Therefore, different reweighting approaches have been developed that either focus on reconstruction of thermodynamic quantities, or additionally perform reweighting of the systems kinetics. In the following, we will discern between these two incentives as “phase-space reweighting” and “dynamic reweighting”. We are providing a condensed overview of both types of reweighting approaches, for a more detailed discussion we refer the interested reader to dedicated reviews and the original literature.^124,127–129^

### 4.1 Phase-space reweighting

Studies that leverage the sampling efficiency of a global biasing potential typically focus on the systems thermodynamics. For methods such as aMD, the most common way to reweight the trajectory data to the unbiased ensemble is to apply a Boltzmann-type reweighting (see the discussion in ref. 84 and 129). The probability of a configuration *r* on the unbiased PES *V*(*r*) is given by,18
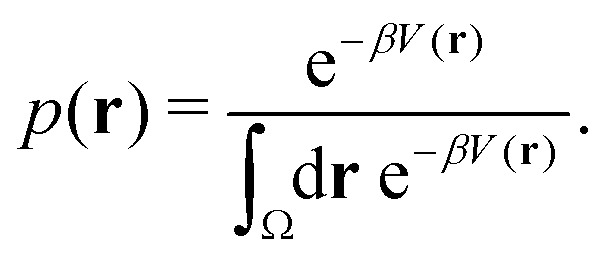


Accordingly, the probability of configuration **r** on the biased PES *Ṽ*(**r**) is,19
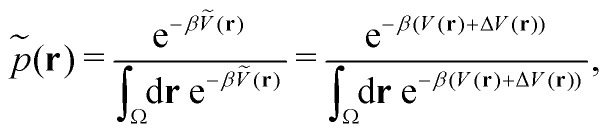
where Δ*V* is the difference between the biased and unbiased PES. Thus, in theory, the probability distribution on the original PES can be reconstructed from the biased PES by multiplication with the Boltzmann factor of the biasing potential,20
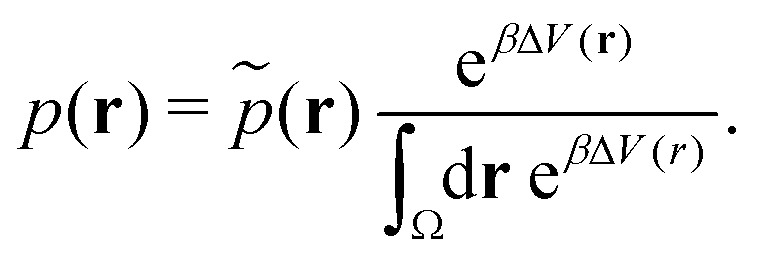


**Fig. 3:**
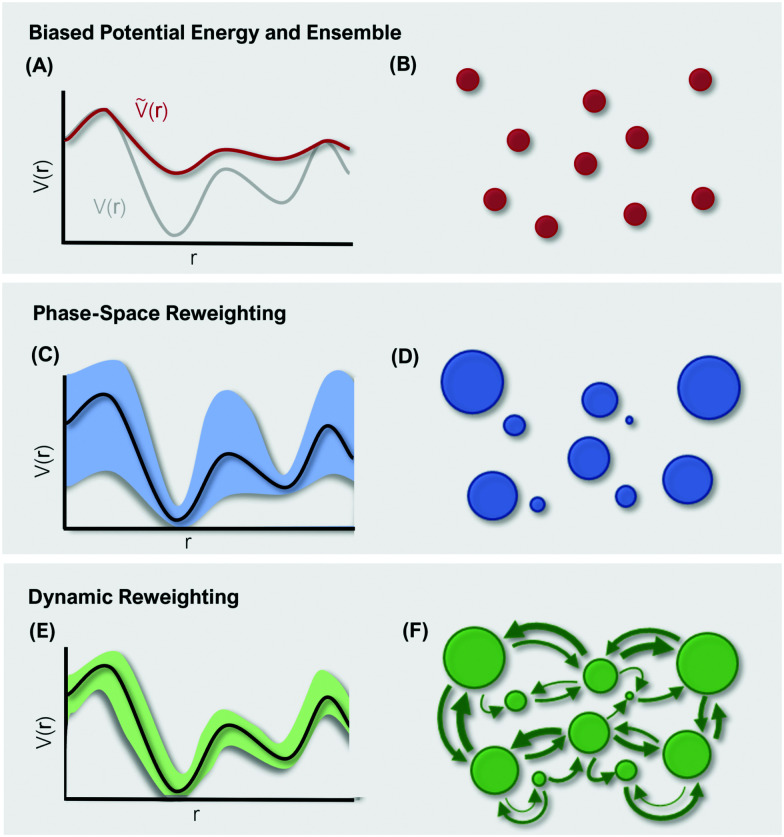
Schematic representation of differences in reweighting a biased PES (A) and a biased ensemble (B). Phase-space reweighting (C and D) reliably recovers the system's thermodynamics, while dynamic reweighting (E and F) additionally provides robust kinetic estimates. The colored bands in (C and E) represent the uncertainty of the reweighted energy profile. The circles in (B, D, and F) represent the system's conformational states, with the size corresponding to their thermodynamic weight.

However, the biasing potential distribution is often very broad in practice, which means that the simulation often spends a substantial amount of time sampling high-energy states. As these high-energy states do not significantly contribute to the ensemble average, the reweighting is effectively based on a relatively small number of frames from the free-energy minima.^124^ Due to the nature of the exponential, the reweighting procedure only works well for comparably narrow distributions of the biasing potential (around 20*k*_B_*T*), and is known to be fairly inaccurate for large systems with broad bias distributions.^129^ This limitation has been bypassed by approximating the exponential term either by a Maclaurin series or cumulant expansion.^129^ The latter has been shown to provide the most reliable results, but is only applicable when the biasing potential follows a Gaussian distribution (which is enforced in the gaMD approach). Still, in particular around the transition regions, the biasing potentials are – by design – comparably small and in practice still vanish in the statistical noise (Fig. 3C). Consequently, relative differences in free energy can typically be reweighted to the unbiased ensemble with robust accuracy, while accurate estimations of barrier heights (*i.e.* kinetics) remain difficult or even inaccessible (Fig. 3).

A widely used approach that has been applied to reweight T-REMD simulations is the weighted histogram analysis method (WHAM).^130^ WHAM was originally defined for a joint analysis of independent simulations in the canonical ensemble and is similar to the Bennet acceptance ratio.^131^ However, advancements of the methodology allow now to generally combine multiple biased ensembles to retrieve a systems unbiased thermodynamics, *e.g.* from T-REMD simulations.^132,133^ Most notably Chodera *et al.*^133^ have introduced an extended WHAM workflow that explicitly considers the time correlation between configurations sampled in each replica. The estimation of the WHAM uncertainty is then corrected by adjusting the true number of independent samples.

The reweighting methods discussed above assume that the input data (*i.e.* the trajectory frames) are uncorrelated samples. However, in biomolecular systems with typically long correlation times this assumption does not necessarily hold.^134^ In particular, simulations of complex biomolecules often do not reversibly sample the equilibrium between different states. Even with enhanced sampling, transitions between conformational states may be observed rarely or only in one direction. Consequently, the proper equilibrium is not sampled and a critical assumption of WHAM is violated.

In summary, phase-space reweighting methods are straight-forward to implement and quickly produce an estimate of the thermodynamics of the system. In recent advances made to GaMD (*i.e.* Ligand-GaMD^135^ and Peptide GaMD^136^), also kinetic information was retrieved directly using Kramer's rate theory. The resulting (un)binding kinetics were found to be on the same order of magnitude with the experimental reference. However, to recover both thermodynamics and kinetics more accurately, the more costly dynamic reweighting methods need to be employed (Fig. 3E and F).

### 4.2 Dynamic reweighting

For unbiased MD simulations, the currently most widely used approach to retrieve robust estimates on a system's kinetics and thermodynamics is to construct a Markov state model (MSM).^137–140^ The same information can be obtained from biased simulation data using dynamic reweighting methods.^141^ The main advantage of MSMs is that the condition of local equilibrium within the simulation data is inherently enforced. The entire approach is based on transitions between states, and in theory only motions that show reversible exchanges between states are considered. Consequently, MSMs provide an ideal theoretical framework for dynamic reweighting strategies.

In recent years, two main classes of dynamic reweighting methods have emerged, path-based and energy-based. The more recently developed path-based reweighting schemes include Weber–Pande reweighting^142^ and Girsanov reweighting.^143^ While their implementation is far from trivial, both have shown reliable results in reweighting dynamics, *e.g.* from umbrella sampling simulations.^142, 143^ The main challenge with path-based dynamic reweighting methods is that they are integrator-dependent, and in practice reweighting needs to be performed on-the-fly and not as a post-processing step.^128^ Energy-based reweighting algorithms, on the other hand, are agnostic to the simulation engine used as they only require information on the bias energy of each analyzed conformation. This less complex handling comes, however, at the price of accuracy – energy-based reweighting does not explicitly account for the possibility that different paths can contribute to the same transition probability. This issue becomes critical whenever the energy profile of the biased PES varies substantially between different pathways. Typically, such a behavior is inherent to pathway-dependent local biasing methodologies and not as pressing with global enhanced sampling techniques. However, in particular in studies on ligand (un)binding or protein folding mechanisms different pathways can lead to substantial deviations in the associated transition rates.^104, 144^ Hence, path-based reweighting might result in more accurate estimates even when a global bias is applied. Prominent examples for energy-based reweighting schemes include the dynamic histogram analysis method (DHAM),^145^ the transition-based reweighting analysis method (TRAM),^134^ and extensions thereof.^146^

While these dynamic reweighting methods were developed and implemented independently by different groups, we were recently able to demonstrate the relationship between them.^128^ In this work by Linker *et al.*, we show that the path-independent DHAM equation is a special case of path-dependent dynamic reweighting. Additionally, we show that both dynamic reweighting families can be connected by introducing a path-correction term to the energy-based method. In doing so, the strongly limiting integrator-dependence of path-based reweighting is omitted, while retaining high accuracy and low parameter sensitivity.^128^

## 5 Conclusion

Enhanced sampling with global biasing functions has massively advanced the field of biomolecular simulations. The constant optimization and extension of promising algorithms has provided our community with the means to simulate large biomolecular complexes, and has opened the door to study conformational changes in the millisecond range. Nevertheless, a common theme for all methodologies is the challenge of choosing optimal parameters with as little pre-processing effort as possible. First attempts towards automated parameter optimization are already being developed, but will need further efficiency improvements for global enhanced sampling to reach its full potential in the study of large biomolecular systems.

As important as the methods for enhanced phase-space exploration are the tools to reweight the biased simulation data to the unbiased canonical ensemble. The development of enhanced sampling techniques goes therefore hand in hand with that of reweighting methods. Over the past decade, multiple algorithms have been proposed that not only recover thermodynamic but also kinetic information from biased simulations. Most of these methods will require further refinement based on “real-world” complex biomolecular systems.

A general question in the application of the methods discussed above is how to validate the insights extracted from the simulations. Direct comparison with experiment is often challenging as techniques such as NMR, cryo-EM, or X-ray crystallography only provide ensemble-averaged data and cannot resolve high-energy states observed in MD simulations. Furthermore, they only provide limited information on a system's kinetics. While NMR (*e.g.* with relaxation dispersion experiments^147,148^) can provide dynamic information, the time scale is typically too long (hundreds of microseconds to milliseconds) even for enhanced sampling MD simulations. One promising strategy to generate reference data for the validation of global enhanced sampling techniques may be the combination of experimental data and MD simulations in integrative structural modeling studies.^4^ Information derived from experiments can be used to augment and guide MD simulations, which in turn provide a structural and dynamic explanation for the measured data.^149,150^

## Conflicts of interest

There are no conflicts to declare.

## Supplementary Material
